# *Rickettsia parkeri* in *Dermacentor parumapertus* Ticks, Mexico

**DOI:** 10.3201/eid2406.180058

**Published:** 2018-06

**Authors:** Sokani Sánchez-Montes, Andrés M. López-Pérez, Carmen Guzmán-Cornejo, Pablo Colunga-Salas, Ingeborg Becker, Jesús Delgado-de la Mora, Jesús D. Licona-Enríquez, David Delgado-de la Mora, Sandor E. Karpathy, Christopher D. Paddock, Gerardo Suzán

**Affiliations:** Universidad Nacional Autónoma de México, Mexico City, Mexico (S. Sánchez-Montes, A.M. López-Pérez, C. Guzmán-Cornejo, P. Colunga-Salas, I. Becker, G. Suzán);; Instituto Nacional de Ciencias Médicas y Nutrición Salvador Zubirán, Mexico City (J. Delgado-de la Mora);; Universidad de Sonora, Sonora, Mexico (J.D. Licona-Enríquez);; Instituto Tecnológico de Sonora, Sonora (D. Delgado-de la Mora);; Centers for Disease Control and Prevention, Atlanta, Georgia, USA (S.E. Karpathy, C. Paddock)

**Keywords:** Rickettsia parkeri, wildlife, ticks, Dermacentor parumapertus, parasites, rickettsiosis, Mexico, vector-borne infections, rickettsia

## Abstract

During a study to identify zoonotic pathogens in northwestern Mexico, we detected the presence of a rickettsial agent in *Dermacentor parumapertus* ticks from black-tailed jackrabbits (*Lepus californicus*). Comparison of 4 gene sequences (*gltA, htrA, ompA,* and *ompB*) of this agent showed 99%–100% identity with sequences of *Rickettsia parkeri*.

*Rickettsia parkeri* is an emerging pathogen that causes a spotted fever group rickettsiosis, transmitted to humans primarily by several species of ticks of the genus *Amblyomma*, including *A*. *maculatum*, *A*. *triste*, *A*. *tigrinum*, and *A*. *ovale*. However, *R. parkeri* has also been detected in other hard ticks, including *A*. *americanum*, *A*. *aureolatum*, *A*. *dubitatum*, *A*. *longirostre*, *A. nodosum*, *A*. *parkeri*, *Ixodes scapularis*, *Rhipicephalus sanguineus*, *Dermacentor parumapertus,* and *D*. *variabilis*, in 8 countries in North (United States), Central (Belize), and South (Colombia, Peru, Brazil, Bolivia, Uruguay, Argentina) America (*1*,*2*) (Figure 1, panel A).

**Figure 1:**
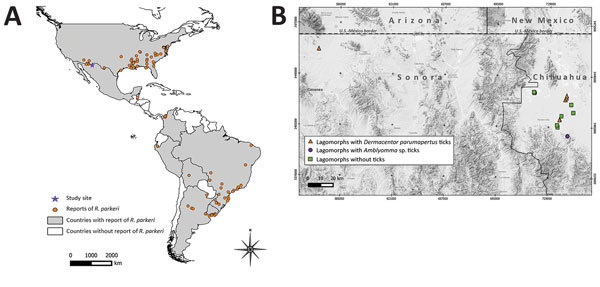
Distribution of *Rickettsia parkeri*. A) Geographic records of *R. parkeri* in the Americas based on reports from the literature. We used a layer of Google Maps (www.google.com/maps/) for the construction of the figure. B) Area of study of *Rickettsia* in Mexico. Squares and triangles represent the sites where lagomorphs carrying ticks were captured.

Recently, a distinct strain of *Rickettsia parkeri,* designated *R. parkeri* Black Gap, was isolated and characterized from the tick *Dermacentor parumapertus* collected from black-tailed jackrabbits in Texas, USA (*3*). This isolate is phylogenetically related to *Rickettsia* sp. strain Atlantic rainforest, a well-known pathogenic lineage of *R. parkeri* associated with a mild rickettsiosis of humans in Brazil (*4*). In addition, the presence of *R. parkeri* in *A. triste* and *A. maculatum* ticks has been confirmed in several locations in Arizona (*5*,*6*). Although *R*. *parkeri*–infected ticks have now been identified in several US states that border Mexico, no studies have demonstrated the presence of *R. parkeri* in ticks in Mexico.

## The Study

We conducted a study to identify zoonotic pathogens in northwestern Mexico; the study area comprised a region within the Janos Biosphere Reserve in Chihuahua state (30°51′50″N, 108°30′09″W) and in the San Pedro River Basin in Sonora state (31°30′90″N, 110°10′70″W) (Figure 1, panel B). The area is in a transition zone between the Sonoran Desert, the Sierra Madre Occidental, and the Chihuahuan Desert and comprises a mosaic of grasslands, mesquite scrublands, and oak forests. During September 2013–September 2014, we sampled lagomorphs in 6 trapping locations (Chihuahuan locations: Casa de Janos, El Cuervo, Monte Verde, Pancho Villa, and Rancho El Uno; Sonoran location: Palmitas). Lagomorphs were live-trapped in box traps and leg-hold traps during a separate study to evaluate *Bartonella* genotypes in wild carnivores (*7*) under permission no. FAUT-0250 of the Secretaría de Medio Ambiente y Recursos Naturales. We identified captured animals as to species, sex, and age (juvenile or adult) using a standard field guide (*8*). We physically restrained the lagomorphs and visually examined them for ticks, removed the ticks manually, and deposited them in cryovials containing 96% ethanol. We then released the hosts in situ. We performed morphological identification of ticks with specialized taxonomic keys (*9*). 

We collected 29 ticks: 23 *D. parumapertus* adults from 21 black-tailed jackrabbits (*Lepus californicus*), 2 *D. parumapertus* adults from 3 white-sided jackrabbits (*Lepus callotis*), and 2 *D. parumapertus* adults and 2 *Amblyomma* sp. nymphs from 4 desert cottontails (*Sylvilagus audubonii*). We deposited 1 female and 2 male *D. parumapertus* ticks in the Colección del Laboratorio de Acarología, Facultad de Ciencias, Universidad Nacional Autónoma de México (UNAM) in Mexico City.

For the remaining specimens, we performed DNA extraction individually using the Chelex100 Chelating Resin (Bio-Rad, Hercules, CA, USA) protocol (*10*). For the initial screening, we amplified a conserved fragment of 805 bp of the *gltA* gene, which is present in all *Rickettsia* species (*11*). We characterized all positive samples by the amplification of 3 additional gene fragments (*ompA*, *ompB*, and *htrA*) using primers and conditions described elsewhere (*11*,*12*). The reaction mixture consisted of 12.5 μL of GoTaq Green Master Mix 2X (Promega, Madison, WI, USA), the corresponding pair of primers (100 ng each), 6.5 μL nuclease-free water, and 100 ng of DNA in a final volume of 25 μL. In all reactions, we included a negative (reaction mix without DNA) and a positive (reaction mix with *Rickettsia lusitaniae* DNA detected in *Ornithodoros yumatensis* from a previous study in southern Mexico [*11*]) control. We sent amplicons of the expected size to Laboratorio de Biología Molecular y de la Salud, UNAM, for purification and sequencing. We compared the sequences obtained with those deposited in GenBank using BLAST (*13*). We deposited sequences recovered in this study in GenBank (accession nos. MG578509–MG578512). We performed global alignments for each gene using the ClustalW algorithm in MEGA 6.0 (https://www.megasoftware.net) and then concatenated them in BioEdit (https://www.mbio/ncsu.edu/BioEdit/bioedit.html). We selected the nucleotide substitution model based on the lowest AICc (Akaike information criterion, corrected). We then generated a maximum likelihood phylogenetic tree with 10,000 bootstrap replications in MEGA 6.0, using the close neighbor interchange method. Gaps were excluded from the analysis.

**Figure 2:**
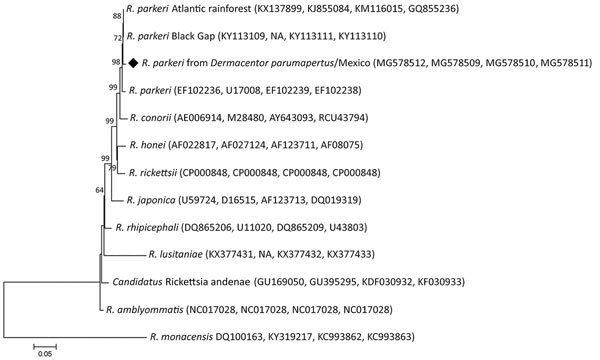
Maximum-likelihood phylogenetic tree generated by using the general time-reversible model using discrete gamma distribution for a total of 2,308 bp of the *gltA*, *htrA*, *ompB*, and *ompA* genes concatenated from a few members of the genus *Rickettsia*. Diamond indicates isolate obtained from ticks in Mexico. Bootstrap values >50% are indicated at the nodes (−In = −6514.38). Numbers in parentheses are GenBank accession numbers. Scale bar indicates nucleotide substitutions per site. NA, not available.

Of the 24 *D. parumapertus* ticks tested, 1 female and 3 males (16.6% total) were positive for the amplification of the gene *gltA*. Neither of the *Amblyomma* nymphal ticks was positive for *Rickettsia* DNA. In addition, we were able to amplify the other 3 genes in the 4 positive samples analyzed. The sequences obtained from the 4 ticks were 100% identical to each other for each corresponding gene. Comparison of 4 gene sequences exhibited 99%–100% identity with the corresponding sequences of *R*. *parkeri* (Table). The final supermatrix consisted of 2,308 bp (731 bp for *gtlA*, 429 bp for *ompA*, 744 bp for *ompB*, and 404 bp for *htrA* genes), with 670 variable sites, 494 singletons, and 176 parsimony informative sites. Additionally, our phylogenetic analysis corroborates the identity of the *Rickettsia* detected, as our sequences and those of references of the different strains of *R. parkeri* form a monophyletic clade with a support value of 98% (Figure 2).

**Table:** Results of analysis of *Rickettsia* sequences recovered from ticks in Mexico to *Rickettsia parkeri* sequences from GenBank*

Gene	Comparison strain (accession no.)	Sequence identity
*gltA*	*R. parkeri* Black Gap (KY124257.1)	731/731, 100%
*ompA*	*R*. *parkeri* RAmova (MF034495.1)	429/429, 100%
	*R*. *parkeri* Atlantic rainforest (KX137902.1)	429/429, 100%
*ompB*	*R*. *parkeri* Black Gap (KY113111.1)	735/744, 98.7%
*htrA*	*R*. *parkeri* (U17008.1)	404/404, 100%

*Analysis conducted using BLAST (*13*).

## Conclusions

We identified DNA of *R. parkeri* in *D. parumapertus* ticks from Chihuahua and Sonora, nearly identical to the Black Gap strain of *R. parkeri* reported previously from *D. parumapertus* ticks from Texas. This tick species is widely distributed across much of northern Mexico, including the states of Baja California, Baja California Sur, Chiapas, Chihuahua, Coahuila, Durango, Hidalgo, Mexico City, San Luis Potosi, and Sonora (*8*,*14*), which suggests that this *Rickettsia* species could be present in other localities in Mexico. *D. parumapertus* ticks have a marked preference for lagomorphs; nonetheless, various other human-biting *Dermacentor* and *Amblyomma* tick species also parasitize lagomorphs, and there is a possibility that these species could acquire *R. parkeri* by co-feeding and subsequently transmit this agent to humans in these regions. The prevalence of *R. parkeri* in ticks from Mexico was consistent with other studies from Texas and Arizona, in which the prevalence ranged from 14% to 24% (*3*,*5*,*6*). We conducted our study in a region of Mexico where spotted fever is endemic; the incidence of spotted fever group rickettsiosis in Chihuahua in 2016 was 0.11 human cases/100,000 inhabitants and that in Sonora was 1.21 human cases/100,000 inhabitants (*15*). All these cases have been attributed to *R. rickettsii*, the cause of Rocky Mountain spotted fever. Although no confirmed cases of disease in humans have been attributed to *R. parkeri* Black Gap infections, previous experimental studies have shown that this isolate could be pathogenic in a guinea pig model (*3*). In addition, its phylogenetic relatedness with *R. parkeri* strain Atlantic rainforest, a well-recognized human pathogen (*4*), will have great meaning for healthcare workers in Mexico. However, further studies should be done to identify the potential of *R. parkeri* Black Gap and the strain of *R. parkeri* we identified as human pathogens. Our findings highlight the importance of studying rickettsial agents in wildlife to identify pathogens of potential public health concern.
